# Clinical Lectures on Medicine

**Published:** 1845-10

**Authors:** Robert J. Graves


*Clinical Lectures on Medicine*, delivered at the Meath Hospital, Dublin. By Robert J. Graves, M.D., M.R.I.A., &c.

Let me now direct your attention to the case of Margaret Lauder, who was admitted a few days ago, for suppression of the catamenia, with obstinate vomiting and persistent gastrodynia. This woman, who is of slender make and rather delicate constitution, has been complaining, more or less, for the last four years, during the greater part of which she has been subject to pain in the stomach, nausea, and vomiting. About five months ago, a new feature was added to her disease; the catamenia became suppressed, and have not appeared since. The attack of vomiting comes on every third or fourth day, sometimes oftener, and is preceded by pain in the region of the stomach, darting across the epigastrium and towards the left shoulder. The fluid ejected from the stomach, which is extremely sour, and as she states, "sets her teeth on edge," is of various colors: green, yellow, gray, or whitish, but never black. There is no remarkable epigastric tenderness, and she finds that during a paroxysm, the violence of the pain is diminished by pressure.

The first question that suggests itself with respect to the nature of this case is, does this long-continued, obstinate, frequently recurring pain of the stomach, nausea, and vomiting, depend on organic disease, or is it the result of mere functional derangement? This is a most important question, and one which you will frequently have occasion to put to yourselves in practice. You will be often consulted in such cases; your examinations will be watched by the patients and their friends with close and painful attention, and your decision awated with all the intense anxiety of minds haunted by the idea of scirrhus. You cannot, gentlemen, be too deeply impressed with the importance attached to such decisions, nor can you apply yourselves too assiduously to the difficult task of making a distinction between organic disease and functional disorder. I am sure I do not exaggerate when I say, that as many professional reputations have been wrecked upon this point, as upon any other connected with the whole range of medical science.

With respect to the present case, my impression is, that there is no organic affection of the stomach. My reasons for coming to this conclusion are these. In the first place, the disease has lasted with more or less violence, for the last four years, and without any remarkable increase. The cause which operated in giving rise to the gastric symptoms three or four years ago, and before the suspicion of organic disease could be entertained, is still in operation, or, in other words, there does not seem to be any recent modification of her ailment, any new change to destroy the identity between her past and present condition. In the next place, the very duration of the complaint is opposed to the idea of its being organic. Had it been organic, it would, in all probability, have terminated fatally before now; or, at least, it would have furnished unequivocal proofs of its existence. Scirrhus of the stomach seldom lasts for four years without exhibiting symptoms of its true nature. Again, the matter from her stomach was never tinged with blood, nor has it ever been of a black color, or consisting of a substance resembling coffee-grounds. Now, I need not tell you, that where organic disease of the stomach produces erosion of its mucous coat, and actual loss of substance, there will be an oozing of blood from the eroded surface, and that this blood will be either thrown up unchanged and of its natural color, or it may remain in the stomach until the fluids of that organ have acted on it, and then it will appear, when ejected, of a dark color, and broken down so as to resemble coffee-grounds. In the next place, there is no remarkable epigastric tenderness, and during the paroxysms, the pain, instead of being increased, is diminished by pressure.

Lastly, the extreme acidity of the fluid ejected from the stomach, is more an indication of functional disorder than of organic disease. I do not lay much stress upon this point, but where a person who has been long subject to pain, nausea, and vomiting, throws up a fluid of an extremely acid taste, the disease is, *in the majority of cases, functional.* How are we to explain this? The acidity of the fluid ejected from the stomach in any considerable quantity depends on derangement of the secreting surface of that organ. As long as the secretion of the stomach is carried on in the natural way, it never becomes a source of annoyance; a certain quantity of acid gastric juice is poured out at certain periods in the day, and these periods correspond in the normal state with the times of taking food, or in other words, this acid juice is only poured out when it is necessary. But in states of deranged action, where the secretion is altered by functional disorder, the stomach pours out this acid juice in excessive quantity. You will find a patient affected in this way vomiting a fluid, which if it fell on powdered chalk or soda, would produce a brisk effervescence, in consequence of the large proportion of acetic and muriatic acid it contains. I have seen cases in which there was absolute corrosion of the teeth from frequent eructation of this extremely acid secretion.

It is clear, that the formation of acid in the stomach is merely the result of an alteration in its secreting powers, and not in consequence of any acetous fermentation of the ingesta. Now, this being the case, if the stomach be extensively diseased from disorganization, the consequence of scirrhous or cancerous ulceration, its secretion will be still further altered, and very frequently in such cases the products of secretion will not be so acid as they are in cases of functional disorder. Organic disease of deranges the functions of the stomach so much, that the secretion of acid is more or less interrupted, and hence we do not find the fluid ejected so sour as it is in functional derangement. I, therefore, look upon the extreme acidity of the fluid thrown up by this woman, as connected with functional disturbance rather than actual disorganization.

I am awaie that the opinion is somewhat different from that advanced by Dr. Osborne in the 21st No. of the *Dublin Journal of Medicine;* Dr. Osborne seems to be of opinion that scirrhus and often cancer of the stomach are the results of functional disorder; there is nothing of which I am more convinced than that they are essentially different. I have known many persons afflicted for years, nay, for life, with the most aggravated form of *acid dyspepsia*, in whom no tendency to organic disease ever displayed itself. That the slow process to which is owing the gradual formation of scirrhus of the stomach will often occasion *acid indigestion*, long before the scirrhus is formed or has reached any degree of maturity, no one will deny. Dr. Osborne has been anticipated in almost all the propositions he has advanced, by Dr. Prus, of Paris, in a book published several years ago, where the same means of averting scirrhus are recommended. I fear that neither Dr. Prus nor Dr. Osborne has as yet discovered this important secret. They certainly deserve our best thanks for the attention they have bestowed on the subject.

Acidity of the stomach is frequently, but not always, accompanied by gastrodynia or pain in the organ. In our patient, violent pain precedes the attacks of vomiting, and is occasionally present during the intervals. There is, however, no connection between pain and acidity of the stomach, for you frequently have severe gastrodynia in cases where the matter ejected has not the slightest acid taste. This is the case in pyrosis, where the fluid thrown up is quite limpid and tasteless. Here it is very probable that the fluid consists chiefly of the secretion of the pancreas.

In inquiring into the symptoms of which dyspeptic patients complain, you will find some with *weak stomachs and strong bowels*, while others have *strong stomachs and weak bowels.* The former complain of acidity, flatulence, distension and uneasiness of the stomach after meals, and have a capricious or indifferent appetite. They suffer much from eating certain articles, and are consequently obliged to be careful in the selection of their food. The digestion thus imperfectly performed in such cases by the stomach, is effected with more energy by the remaining portion of the intestinal canal, and hence the fæcal mass is quite healthy, and contains no unused alimentary matter. In order to enable the bowels to perform more efficiently the supplemental digestion, so necessary where the principal agent, the stomach, has left so much undone, the alimentary mass is detained longer than natural in the intestinal tube. In such persons, costiveness or a slow action of the bowels, is essential for the more complete solution and absorption of the nutritive portion of food which the stomach had, as it were, neglected. If by means of medicine or diet you promote a quick action of the bowels, and a speedy defecation in such persons, you impair the supplemental digestion, and injure rather than serve your patients. In cases of this description we frequently observe, that although great disturbance of the stomach ensues after meals, yet there is little or no emaciation or loss of strength; the sufferers are often of robust, phlegmatic habit. The theory of digestion which attributed to the stomach the office of chymification—a step necessary for the more advanced process of chylification supposed to take place in the small intestines—this theory will not explain the phenomena observed in the above cases, for nutrition must, according to this theory, be impaired exactly in proportion to the degree in which the stomachic digestion fails. Here the theory of digestion which rests upon the authority of Tiedemann and Gmelin, comes opportunely to our aid. According to their experiments it appears that the nutritive efficiency of digestion depends on the solution and subsequent absorption of the soluble and nourishing portions of the alimentary mass. Now, solution and absorption are performed by the whole extent of the alimentary canal, but most energetically by the stomach and cæcum or commencement of the large gut, which two parts are the chief secretors of the acid solvents. From this it follows, that when the stomach fails to perform the whole of its duty, its deficiencies may be supplied by the intestines. The history of intestinal fiistula where the excrementitious matter is voided through an opening in the small intestines, proves that a man may live without the discharge of the functions which the large intestines are intended to perform on the alimentary mass. Again, those rarer cases of stomachic fistula which have been observed, almost suggest the possibility of life and nutrition being sustained, although imperfectly, without the aid of the stomach; for we cannot believe that this organ performs its functions with anything like natural energy, when it is placed in so unnatural a situation, and has suffered so much from previous disease and morbid alteration of structure. What is wanting in such cases to prove that nutrition may be carried on, nearly altogether independently of the stomach, is supplied by other organic diseases of that organ. Thus, in the case of Napoleon Bonaparte: the body was by no means wasted, on the contrary, there was a great accumulation of fat; "the fat was upwards of one inch thick over the sternum, and one inch and a half over the abdomen; upon opening the abdomen, the omentum was found remarkably fat, and on exposing the stomach, that viscus was found the seat of extensive disease; strong adhesions connected the whole superior surface, particularly about the pyloric extremity to the concave surface of the left lobe of the liver, and on separating these, an ulcer, which penetrated the coats of the stomach, was discovered one inch from the pylorus, sufficient to allow the passage of the little finger. The internal surface of the stomach in nearly its whole extent was a mass of cancerous disease, or scirrhous portions advancing to cancer; this was particularly noticed near the pylorus; the cardiac extremity, for a small space near the termination of the œsophagus, was the only part appearing in a healthy condition."

Now, gentlemen, in this and similar cases, it is evident that no part of the digestive function had been performed by the stomach, probably for several weeks, or even longer, before death, and yet the body was so sufficiently nourished by the supplemental action of the small intestines, that we are almost tempted to believe that if by a miracle the cancerous mass including the whole stomach had been removed, and had been replaced by a portion of the duodenum, the individual might have lived for a time! But to return to our subject—where the stomach is strong, but the intestines weak, there is generally an excellent appetite, and the patient is a great eater—he never suffers from distension of the stomach, uneasy feelings, acidity, flatulence or heart-burn after meals, however he may have indulged; he boasts, and justly, that nothing ever disagrees with his stomach. He is nevertheless lean and ill-thriven; the reason is because his bowels do not possess the same energy with the stomach, and consequently do not perform their part of the business of digestion well; this is evident from the frequent and sudden attacks of looseness to which such persons are liable; their discharges are irregular, but on the whole, the quantity of matter discharged *per anum* is much greater than it ought to be; they seldom pass solid healthy colored stools—there is evidence of a hurried secretion of bile, and a too rapid passage of the alimentary mass through the intestines. The fæces are therefore during their occasional attacks of indigestion crude and semi-digested, and almost constantly fluid or semi-fluid; such persons have two or three bilious, loose motions every day, and generally enjoy in other respects tolerably good health.

Having pointed out these varieties of weak digestion, varieties which have scarcely been noticed by authors, but which nevertheless deserve especial attention, I shall conclude by observing that the distinction between organic disease and functional disorder of the stomach is attended with extreme difficulty, and is to be drawn not from any one symptom, but from an attentive consideration of the history of the case, and the effects of remedies.

I have seen cases of indigestion, vomiting, and extreme emaciation continue for month after month, until every medical attendant of the patient believed that death from organic disease was at hand, and yet the patient perfectly recovered; on the other hand I have seen the stomach ailment trifling when organic disease was extensive. I am anxious to impress this upon your minds lest you may be misled by anything I have said in a previous part of the lecture, and lest you should think that I attach undue importance to any one symptom, as for instance, the morbid secretion of acid by the stomach. Dr. Osborne seems to look upon this rather as a symptom of commencing scirrhus; in this I cannot agree with him; probably we are both wrong; it probably cannot be relied on as characteristic either of functional disorder or of organic disease.—*Medical Times.—Medical News and Library.*

